# (*E*,*E*)-4,4′-Dichloro-2,2′-[azinobis(phenyl­methyl­idyne)]diphenol

**DOI:** 10.1107/S1600536809041427

**Published:** 2009-10-17

**Authors:** Jian-Guo Chang

**Affiliations:** a Department of Materials Science and Chemical Engineering, Taishan University, 271021 Taian,Shandong, People’s Republic of China

## Abstract

The title compound, C_26_H_18_Cl_2_N_2_O_2_, was synthesized by the reaction of (5-chloro-2-hydroxy­phen­yl)(phen­yl)methanone with hydrazine hydrate. The mol­ecule possesses a crystallographically imposed centre of symmetry at the mid-point of the N—N bond. The conformation of the mol­ecule is stabilized by an intra­molecular O—H⋯N hydrogen bond.

## Related literature

For further details of the chemistry of the title compound, see: Glaser *et al.* (1995 ▶); Hunig *et al.* (2000 ▶). For similar structures, see: Kundu *et al.* (2005 ▶); Chang *et al.* (2007 ▶); Kesslen *et al.* (1999 ▶).
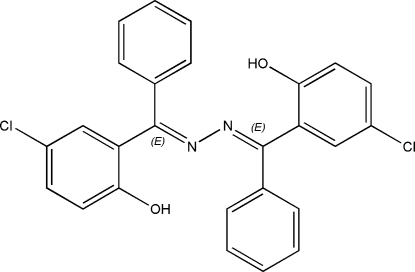


## Experimental

### 

#### Crystal data

C_26_H_18_Cl_2_N_2_O_2_
*M* *_r_* = 461.32
Orthorhombic, 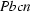

*a* = 13.1622 (11) Å
*b* = 10.6184 (9) Å
*c* = 16.0671 (13) Å
*V* = 2245.6 (3) Å^3^
*Z* = 4
Mo *K*α radiation
μ = 0.32 mm^−1^
*T* = 295 K
0.21 × 0.19 × 0.15 mm

#### Data collection

Bruker APEXII CCD area-detector diffractometer
Absorption correction: multi-scan (*SADABS*; Sheldrick, 2003 ▶) *T*_min_ = 0.925, *T*_max_ = 0.960
11062 measured reflections
1995 independent reflections
1448 reflections with *I* > 2σ(*I*)
*R*_int_ = 0.033

#### Refinement

*R*[*F* ^2^ > 2σ(*F* ^2^)] = 0.045
*wR*(*F* ^2^) = 0.163
*S* = 1.00
1995 reflections
146 parameters
H-atom parameters constrained
Δρ_max_ = 0.23 e Å^−3^
Δρ_min_ = −0.41 e Å^−3^

### 

Data collection: *APEX2* (Bruker, 2005 ▶); cell refinement: *SAINT* (Bruker, 2005 ▶); data reduction: *SAINT*; program(s) used to solve structure: *SHELXS97* (Sheldrick, 2008 ▶); program(s) used to refine structure: *SHELXL97* (Sheldrick, 2008 ▶); molecular graphics: *SHELXTL* (Sheldrick, 2008 ▶); software used to prepare material for publication: *SHELXTL*.

## Supplementary Material

Crystal structure: contains datablocks global, I. DOI: 10.1107/S1600536809041427/pk2195sup1.cif

Structure factors: contains datablocks I. DOI: 10.1107/S1600536809041427/pk2195Isup2.hkl

Additional supplementary materials:  crystallographic information; 3D view; checkCIF report

## Figures and Tables

**Table 1:** Hydrogen-bond geometry (Å, °)

*D*—H⋯*A*	*D*—H	H⋯*A*	*D*⋯*A*	*D*—H⋯*A*
O1—H1⋯N1	0.82	1.85	2.572 (3)	145

**Fig. 1.**
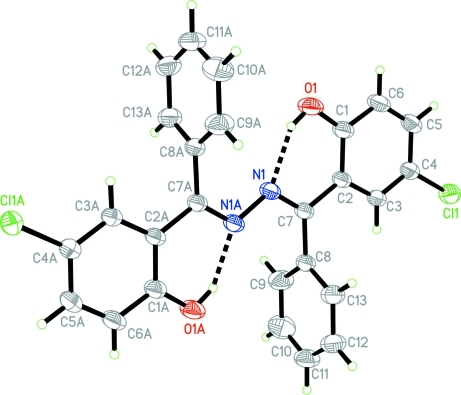
The molecular structure of compound, showing the atom-numbering scheme. Displacement ellipsoids are drawn at the 30% probability level. Dashed lines show intramolecular hydrogen bonds.

## supplementary crystallographic information

### Comment

Recently, a number of azine compounds containing both a diimine linkage and N—N bonding have been investigated in terms of their crystallography and coordination chemistry (Kundu *et al.*, 2005; Kesslen *et al.*, 1999; Chang *et al.*, 2007;).  As an extension of work on the structural characterization of azine derivatives, the title compound was synthesized and its crystal structure is reported here.

In the title compound, there is a crystallographic centre of symmetry at the midpoint of the N—N bond (Fig. 1.). The molecule displays an (E, E) conformation with respect to the C7=N1 and its symmetry related *c*7*a*=N1a double bond (Fig. 1.). This configuration agrees with those commonly found in similar compounds (Glaser *et al.*, 1995; Hunig *et al.*, 2000). The benzene rings, C1—C6(A), C8—C13(B) make dihedral angles of 85.26 (10)°. The conformation of the molecule is stabilized by intramolecular O—H···N hydrogen bonds. (Table 1. and Fig. 1).

### Experimental

An ethanol solution (50 ml) of hydrazine (0.02 mol) and (5-chloro-2-hydroxyphenyl)(phenyl)methanone (0.04 mol) was refluxed and stirred for 6 h; the mixture was cooled and the resulting solid product, was collected by filtration. Crystals suitable for single-crystal X-ray diffraction were grown by slow evaporation of a solution in acetone.

### Refinement

All H atoms were positioned geometrically and treated as riding on their parent atoms, with C—H(aromatic) = 0.93 Å, O—H = 0.82 Å and with *U*_iso_(H) =1.5*U*_eq_(O) and 1.2*U*_eq_(C_ar_).

### Crystal data

**Table:**

C_26_H_18_Cl_2_N_2_O_2_	*F*(000) = 952
*M**_r_* = 461.32	*D*_x_ = 1.365 Mg m^−^^3^
Orthorhombic, *P**b**c**n*	Mo *K*α radiation, λ = 0.71073 Å
Hall symbol: -P 2n 2ab	Cell parameters from 2411 reflections
*a* = 13.1622 (11) Å	θ = 2.5–24.9°
*b* = 10.6184 (9) Å	µ = 0.32 mm^−^^1^
*c* = 16.0671 (13) Å	*T* = 295 K
*V* = 2245.6 (3) Å^3^	Plate, yellow
*Z* = 4	0.21 × 0.19 × 0.15 mm

### Data collection

**Table:**

Bruker APEXII CCD area-detector diffractometer	1995 independent reflections
Radiation source: fine-focus sealed tube	1448 reflections with *I* > 2σ(*I*)
graphite	*R*_int_ = 0.033
φ and ω scans	θ_max_ = 25.1°, θ_min_ = 2.5°
Absorption correction: multi-scan (*SADABS*; Sheldrick, 2003)	*h* = −7→15
*T*_min_ = 0.925, *T*_max_ = 0.960	*k* = −12→12
11062 measured reflections	*l* = −18→19

### Refinement

**Table:**

Refinement on *F*^2^	Primary atom site location: structure-invariant direct methods
Least-squares matrix: full	Secondary atom site location: difference Fourier map
*R*[*F*^2^ > 2σ(*F*^2^)] = 0.045	Hydrogen site location: inferred from neighbouring sites
*wR*(*F*^2^) = 0.163	H-atom parameters constrained
*S* = 1.00	*w* = 1/[σ^2^(*F*_o_^2^) + (0.113*P*)^2^ + 0.120*P*] where *P* = (*F*_o_^2^ + 2*F*_c_^2^)/3
1995 reflections	(Δ/σ)_max_ < 0.001
146 parameters	Δρ_max_ = 0.23 e Å^−^^3^
0 restraints	Δρ_min_ = −0.41 e Å^−^^3^

### Special details

**Table:**

Geometry. All e.s.d.'s (except the e.s.d. in the dihedral angle between two l.s. planes) are estimated using the full covariance matrix. The cell e.s.d.'s are taken into account individually in the estimation of e.s.d.'s in distances, angles and torsion angles; correlations between e.s.d.'s in cell parameters are only used when they are defined by crystal symmetry. An approximate (isotropic) treatment of cell e.s.d.'s is used for estimating e.s.d.'s involving l.s. planes.
Refinement. Refinement of *F*^2^ against ALL reflections. The weighted *R*-factor *wR* and goodness of fit *S* are based on *F*^2^, conventional *R*-factors *R* are based on *F*, with *F* set to zero for negative *F*^2^. The threshold expression of *F*^2^ > σ(*F*^2^) is used only for calculating *R*-factors(gt) *etc*. and is not relevant to the choice of reflections for refinement. *R*-factors based on *F*^2^ are statistically about twice as large as those based on *F*, and *R*- factors based on ALL data will be even larger.

### Fractional atomic coordinates and isotropic or equivalent isotropic displacement parameters (Å^2^)

**Table:**

	*x*	*y*	*z*	*U*_iso_*/*U*_eq_	
Cl1	0.23969 (7)	0.00971 (8)	0.37894 (5)	0.0751 (4)	
O1	0.0498 (2)	−0.25287 (17)	0.10094 (15)	0.0776 (7)	
H1	0.0336	−0.2049	0.0632	0.116*	
N1	0.01697 (18)	−0.03788 (18)	0.03234 (13)	0.0534 (6)	
C1	0.0915 (2)	−0.1871 (2)	0.16297 (18)	0.0584 (8)	
C2	0.09948 (19)	−0.0543 (2)	0.16200 (16)	0.0487 (7)	
C3	0.1464 (2)	0.0039 (2)	0.22956 (15)	0.0502 (7)	
H3	0.1534	0.0911	0.2300	0.060*	
C4	0.1822 (2)	−0.0646 (2)	0.29475 (16)	0.0555 (7)	
C5	0.1737 (2)	−0.1941 (3)	0.29588 (19)	0.0671 (8)	
H5	0.1983	−0.2402	0.3408	0.081*	
C6	0.1291 (3)	−0.2536 (3)	0.2306 (2)	0.0709 (9)	
H6	0.1235	−0.3409	0.2312	0.085*	
C7	0.06111 (19)	0.0215 (2)	0.09230 (15)	0.0468 (6)	
C8	0.0749 (2)	0.1609 (2)	0.09250 (16)	0.0503 (7)	
C9	−0.0003 (3)	0.2398 (3)	0.1189 (2)	0.0801 (10)	
H9	−0.0609	0.2068	0.1391	0.096*	
C10	0.0135 (3)	0.3694 (3)	0.1158 (3)	0.1007 (13)	
H10	−0.0377	0.4230	0.1342	0.121*	
C11	0.1022 (3)	0.4180 (3)	0.0857 (2)	0.0905 (11)	
H11	0.1108	0.5048	0.0827	0.109*	
C12	0.1777 (3)	0.3402 (3)	0.0602 (2)	0.0782 (10)	
H12	0.2384	0.3737	0.0405	0.094*	
C13	0.1644 (2)	0.2118 (3)	0.06340 (19)	0.0660 (8)	
H13	0.2164	0.1587	0.0457	0.079*	

### Atomic displacement parameters (Å^2^)

**Table:**

	*U*^11^	*U*^22^	*U*^33^	*U*^12^	*U*^13^	*U*^23^
Cl1	0.0985 (7)	0.0654 (6)	0.0615 (6)	−0.0123 (4)	−0.0284 (4)	0.0114 (3)
O1	0.124 (2)	0.0360 (11)	0.0729 (14)	−0.0034 (11)	−0.0286 (14)	0.0010 (9)
N1	0.0740 (15)	0.0362 (11)	0.0500 (12)	0.0003 (10)	−0.0115 (10)	0.0046 (9)
C1	0.0757 (19)	0.0383 (14)	0.0612 (17)	0.0019 (12)	−0.0088 (14)	0.0026 (12)
C2	0.0576 (15)	0.0352 (12)	0.0533 (14)	0.0035 (11)	−0.0037 (11)	0.0052 (11)
C3	0.0596 (15)	0.0379 (12)	0.0531 (15)	−0.0002 (11)	−0.0045 (11)	0.0052 (11)
C4	0.0639 (16)	0.0462 (14)	0.0565 (15)	0.0000 (12)	−0.0074 (13)	0.0065 (12)
C5	0.084 (2)	0.0506 (16)	0.0673 (18)	0.0046 (15)	−0.0175 (15)	0.0158 (14)
C6	0.099 (2)	0.0402 (14)	0.0733 (19)	0.0036 (14)	−0.0188 (17)	0.0107 (13)
C7	0.0564 (15)	0.0362 (13)	0.0477 (13)	0.0009 (10)	−0.0035 (11)	0.0037 (10)
C8	0.0636 (16)	0.0387 (13)	0.0486 (13)	−0.0017 (12)	−0.0098 (12)	0.0031 (11)
C9	0.080 (2)	0.0472 (17)	0.113 (3)	0.0045 (15)	0.0075 (18)	−0.0080 (16)
C10	0.112 (3)	0.057 (2)	0.133 (3)	0.022 (2)	−0.006 (2)	−0.015 (2)
C11	0.128 (3)	0.0418 (18)	0.102 (3)	−0.0125 (19)	−0.032 (2)	0.0059 (17)
C12	0.104 (2)	0.0579 (19)	0.0724 (19)	−0.0258 (18)	−0.0146 (18)	0.0126 (16)
C13	0.0825 (19)	0.0504 (16)	0.0653 (17)	−0.0083 (14)	−0.0043 (15)	0.0053 (13)

### Geometric parameters (Å, °)

**Table:**

Cl1—C4	1.739 (3)	C6—H6	0.9300
O1—C1	1.335 (3)	C7—C8	1.491 (4)
O1—H1	0.8200	C8—C9	1.365 (4)
N1—C7	1.290 (3)	C8—C13	1.378 (4)
N1—N1^i^	1.388 (4)	C9—C10	1.388 (4)
C1—C6	1.387 (4)	C9—H9	0.9300
C1—C2	1.414 (4)	C10—C11	1.365 (5)
C2—C3	1.394 (4)	C10—H10	0.9300
C2—C7	1.469 (3)	C11—C12	1.355 (5)
C3—C4	1.360 (3)	C11—H11	0.9300
C3—H3	0.9300	C12—C13	1.376 (4)
C4—C5	1.381 (4)	C12—H12	0.9300
C5—C6	1.359 (4)	C13—H13	0.9300
C5—H5	0.9300		
			
C1—O1—H1	109.5	N1—C7—C8	122.8 (2)
C7—N1—N1^i^	114.9 (2)	C2—C7—C8	120.0 (2)
O1—C1—C6	117.7 (2)	C9—C8—C13	119.0 (3)
O1—C1—C2	122.9 (2)	C9—C8—C7	121.5 (3)
C6—C1—C2	119.4 (3)	C13—C8—C7	119.5 (2)
C3—C2—C1	117.8 (2)	C8—C9—C10	120.2 (4)
C3—C2—C7	120.2 (2)	C8—C9—H9	119.9
C1—C2—C7	122.0 (2)	C10—C9—H9	119.9
C4—C3—C2	121.1 (2)	C11—C10—C9	120.0 (4)
C4—C3—H3	119.5	C11—C10—H10	120.0
C2—C3—H3	119.5	C9—C10—H10	120.0
C3—C4—C5	121.0 (2)	C12—C11—C10	120.2 (3)
C3—C4—Cl1	120.50 (19)	C12—C11—H11	119.9
C5—C4—Cl1	118.5 (2)	C10—C11—H11	119.9
C6—C5—C4	119.2 (2)	C11—C12—C13	120.0 (3)
C6—C5—H5	120.4	C11—C12—H12	120.0
C4—C5—H5	120.4	C13—C12—H12	120.0
C5—C6—C1	121.5 (2)	C12—C13—C8	120.6 (3)
C5—C6—H6	119.3	C12—C13—H13	119.7
C1—C6—H6	119.3	C8—C13—H13	119.7
N1—C7—C2	117.2 (2)		
			
O1—C1—C2—C3	−178.7 (3)	C1—C2—C7—N1	1.8 (4)
C6—C1—C2—C3	0.8 (4)	C3—C2—C7—C8	1.7 (4)
O1—C1—C2—C7	0.6 (4)	C1—C2—C7—C8	−177.6 (2)
C6—C1—C2—C7	−179.9 (3)	N1—C7—C8—C9	83.9 (4)
C1—C2—C3—C4	−0.8 (4)	C2—C7—C8—C9	−96.8 (3)
C7—C2—C3—C4	179.9 (2)	N1—C7—C8—C13	−94.4 (3)
C2—C3—C4—C5	0.4 (4)	C2—C7—C8—C13	84.9 (3)
C2—C3—C4—Cl1	−179.5 (2)	C13—C8—C9—C10	0.5 (5)
C3—C4—C5—C6	0.2 (5)	C7—C8—C9—C10	−177.8 (3)
Cl1—C4—C5—C6	−180.0 (3)	C8—C9—C10—C11	0.4 (6)
C4—C5—C6—C1	−0.2 (5)	C9—C10—C11—C12	−1.2 (6)
O1—C1—C6—C5	179.2 (3)	C10—C11—C12—C13	1.0 (5)
C2—C1—C6—C5	−0.3 (5)	C11—C12—C13—C8	−0.1 (5)
N1^i^—N1—C7—C2	−179.4 (3)	C9—C8—C13—C12	−0.6 (4)
N1^i^—N1—C7—C8	0.0 (4)	C7—C8—C13—C12	177.7 (3)
C3—C2—C7—N1	−178.9 (2)		

Symmetry codes: (i) −*x*, −*y*, −*z*.

### Hydrogen-bond geometry (Å, °)

**Table:**

*D*—H···*A*	*D*—H	H···*A*	*D*···*A*	*D*—H···*A*
O1—H1···N1	0.82	1.85	2.572 (3)	145

## References

[bb1] Bruker (2005). *APEX2* and *SAINT* Bruker AXS Inc., Madison, Wisconsin, USA.

[bb2] Chang, J.-G., He, G.-F. & Li, Y.-F. (2007). *Acta Cryst.* E**63**, o3982.

[bb3] Glaser, R., Chen, G. S., Anthamatten, M. & Barnes, C. L. (1995). *J. Chem. Soc. Perkin Trans. 2*, pp. 1449–1458.

[bb4] Hunig, S., Kemmer, M. & Wenner, H. (2000). *Chem. Eur. J.* **6**, 2618–2632. 10.1002/1521-3765(20000717)6:14<2618::aid-chem2618>3.0.co;2-w 10961407

[bb5] Kesslen, E. C., Euler, W. B. & Foxman, B. M. (1999). *Chem. Mater.* **11**, 336–340.

[bb6] Kundu, N., Chatterjee, P. B., Chaudhury, M. & Tiekink, E. R. T. (2005). *Acta Cryst.* E**61**, m1583–m1585.

[bb7] Sheldrick, G. M. (2003). *SADABS* Bruker AXS Inc., Madison, Wisconsin, USA.

[bb8] Sheldrick, G. M. (2008). *Acta Cryst.* A**64**, 112–122. 10.1107/S0108767307043930 18156677

